# Two Novel Cloud-Masking Algorithms Tested in a Tropical Forest Setting Using High-Resolution NICFI-Planet Basemaps

**DOI:** 10.3390/s25247559

**Published:** 2025-12-12

**Authors:** K. M. Ashraful Islam, Shahriar Abir, Robert Kennedy

**Affiliations:** 1 College of Earth, Ocean, and Atmospheric Sciences, Oregon State University, Corvallis, OR 97331, USA; 2 Department of Urban and Regional Planning, Chittagong University of Engineering and Technology, Chattogram 4349, Bangladesh

**Keywords:** NICFI-Planet, cloud, high resolution, Google Earth Engine (GEE), mangrove, remote sensing

## Abstract

High-resolution NICFI-Planet image collection on Google Earth Engine (GEE) promises fine-scale tropical forest monitoring, but persistent cloud covers, shadows, and haze undermine its value. Here, we present two simple, fully reproducible cloud-masking algorithms. We introduce (A) a Blue and Near-Infrared threshold and (B) a Sentinel-2-derived statistical thresholding approach that sets per-band cutoffs. Both are implemented end-to-end in GEE for operational use. The algorithms were first developed, tuned, and evaluated in the Sundarbans (Bangladesh) using strongly contrasting dry- and monsoon-season scenes. To assess their broader utility, we additionally tested them in two independent deltaic mangrove systems, namely, the Bidyadhari Delta in West Bengal, India, and the Ayeyarwady Delta in Myanmar. Across all sites, Algorithm B consistently removes the largest share of cloud and bright-water pixels but tends to over-mask haze and low-contrast features. Algorithm A retains more usable pixels; however, its aggressiveness is region-dependent. It appears more conservative in the Sundarbans but noticeably more over-inclusive in the India and Myanmar scenes. A Random Forest classifier provided map offers a useful reference but the model is dependent on the quantity and quality of labeled samples. The novelty of the algorithms lies in their design specifically for NICFI-Planet basemaps and their ability to operate without labeled samples. Because they rely on simple, fully shareable GEE code, they can be readily applied in regions in a consistent manner. These two algorithms offer a pragmatic operational pathway: apply them as a first-pass filter keeping in mind that its behavior may vary across environments.

## 1. Introduction

During the past two decades, remote sensing technology has been increasingly adopted for tropical forest and wetland research [1,2]. Remote sensing with optical satellites offers superior spatial detail for monitoring land covers, including tropical forests and wetlands [3,4,5], but is frequently impeded by persistent cloud cover [6,7]. An extensive corpus of the literature used optical sensors of MODIS, Landsat and Sentinel-2 satellites and employed mature cloud-masking algorithms to mitigate this challenge and maintain consistent, moderate-resolution monitoring [8,9,10]. However, as these sensors provide moderate spatial resolution (which can miss fine-scale or fragmented forest patches), it thus motivates the use of finer-resolution data. The Norway’s International Climate and Forests Initiative (NICFI) Satellite Data Program offered researchers and policymakers PlanetScope imagery (also known as NICFI-Planet) with fine spatial resolution (4.77 m) [11], and it can be used to monitor small-scale topical forests and wetlands [12,13,14,15]. Recently, NICFI-Planet data was employed for local-scale tropical forest mapping, which aligned closely with national-scale basemaps [16]. However, currently, there is no straightforward, automated cloud-masking algorithm specifically designed for Planet basemaps in tropical forest settings. Many studies using these images for tropical forest monitoring disregard entire scenes when persistent cloud cover is present [13,14,15]. This hinders precise quantification of tropical forest and wetland change at granular scales in cloud-prone regions.

Cloud and cloud-shadow detection is a well-established topic in remote sensing, with numerous algorithms developed for multispectral satellite systems. Among the most widely used approaches are Fmask [17], MAJA [18], and s2cloudless [19]. Time-series screening methods [20] further improve detection of frequently cloudy tropical regions by leveraging multi-temporal images. Most of these methods depend on Short-Wave Infrared (SWIR), cirrus, and thermal infrared bands to differentiate clouds, haze, water, and bright surfaces. However, these approaches cannot be directly applied to NICFI-Planet basemaps, which provide only four bands (Red, Green, Blue and Near-Infrared; RGB–NIR) at 4.77 m resolution. The absence of SWIR and thermal information limits the applicability of conventional algorithms. In our preliminary observation, we also found that NICFI-Planet basemaps also often retain a thin haze, fragmented cloud edges, and ambiguous transition lines in humid tropical forests. Therefore, despite extensive prior work, there remains no dedicated automated cloud-masking method optimized for RGB–NIR NICFI-Planet mosaics.

To fill this methodological gap, we propose two automatic, unsupervised cloud-masking algorithms based on simple thresholding approaches. To evaluate their effectiveness, we applied both methods to NICFI-Planet basemaps of a tropical mangrove forest in Bangladesh, where cloud cover was visually apparent. We assessed the ability of each algorithm to accurately detect cloud-covered pixels and visually compared their performance against a supervised machine learning-based method. Our goal was to develop a straightforward, easy-to-implement solution that requires no manual intervention. Such unsupervised methods are valuable for masking clouds in large, multi-temporal NICFI-Planet images, where manual labeling or training-based approaches are impractical. Hence, the current endeavor provides a low-overhead, easily implementable solution for masking clouds, which will aid in consistent and timely monitoring of tropical forest and wetland dynamics without the need for complex analysis or external software.

## 2. Methods, Test Site, and Data

### 2.1. Test Site and Data

**Figure 1:**
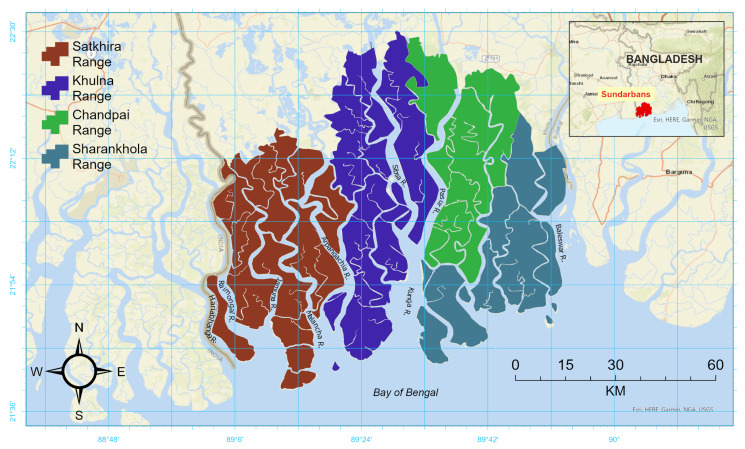
The Sundarbans mangrove forest and the managed forest ranges of Bangladesh Forest Department are shown on the study area map. Polygons obtained from geoBoundaries [23] are used to delineate the forest range boundaries, which are displayed over ESRI World Street Map [24].

The tropical Sundarbans mangrove forest in Bangladesh served as a case study for testing our cloud-masking algorithms (Figure 1). It is the largest continuous chunk of national mangrove forest in the world, covering approximately 620,000 hectares [21] and managed by Bangladesh Forest Department. The climate is characterized by a monsoon season from June to September, which accounts for approximately 71% of annual rainfall, while the postmonsoon and premonsoon (winter) periods are dry [22]. Less than 100 mm of total precipitation is observed in the months from November to March.

The NICFI-Planet basemap mosaics are intended for the detection of deforestation and degradation in tropical areas [11]. The mosaics have a spatial resolution of 4.77 m and comprise four spectral bands: Red (R), Green (G), Blue (B), and Near-Infrared (NIR) [25]. Atmospheric corrections for the NICFI mosaics utilize seasonal models developed from Landsat data [11]. Cloud masking has been pre-applied to the data for users [11]. However, when all available scenes for a location consist of cloudy or otherwise unusable pixels, the highest-ranked cloudy pixels are incorporated to fulfill coverage requirements [11]. Despite NICFI-Planet’s cloud filtering efforts, residual cloud cover remains a challenge in tropical regions [13,14,15]. From September 2020, the mosaics are provided on a monthly basis, and is available on Google Earth Engine (GEE) [26]. For this study, NICFI-Planet data was retrieved from GEE.

We also utilized Sentinel-2 scenes alongside Planet-NICFI basemaps to create reliable, cloud-free statistical baselines for threshold derivation for one of our algorithms [4]. Sentinel-2 surface reflectance products were chosen due to their 10 m spatial resolution in the Blue (B2), Green (B3), Red (B4), and Near-infrared (B8) bands, consistent global radiometry, and availability in GEE archive [4].

### 2.2. Algorithm A: Cloud and Shadow Detection Using B and NIR Bands

A simple masking algorithm was created using GEE to reduce the effect of clouds, cloud shadows, and anomalous pixels by applying specific thresholds to the B and NIR bands. B and NIR bands of Landsat-8, Seninel-2, GaoFen-1, and WorldView-2 have been found to be effective in masking cloud and cloud shadow [8,27,28]. Therefore, this study examined the sensitivity of reflectance values of the NICFI-Planet images in the B and NIR bands to cloudy and shadowed pixels using a combination of threshold values. A final threshold value of B: 0.09 and NIR: 0.35 was selected (the details are described in Section 3 Results and Discussion) for atmospheric filtering based on their observed high sensitivity to cloud cover and shadows. The masks created by both bands are combined and a final mask is created that retains only cloud-free and shadow-free pixels. The filtering mask M(B,N) can be expressed as

$$M\left(B,N\right)=\left\{\begin{array}{cc} 1 & \mathrm{if}\;B\leq t_{B}\;\mathrm{AND}\;N\leq t_{NIR}\\ 0 & \mathrm{otherwise} \end{array}\right.$$

where

-     *B*: Reflectance value of the Blue band.
-     *N*: Reflectance value of the Near-Infrared band.
-     $t_{B}$: Threshold value for the Blue band.
-     $t_{NIR}$: Threshold value for the Near-Infrared band.

The output is 1 if the pixel is valid (passes both the cloud and shadow tests), and 0 otherwise. This mask is then applied to the original image, removing any pixels identified as cloudy or shadowed.

### 2.3. Algorithm B: Statistical Thresholding Using Sentinel-2 Bands

Using GEE, we generated 10 m cloud-free Sentinel-2 median mosaics for the study region to establish a statistical baselines. There is substantial precedent for using Sentinel-2 surface reflectance as a statistical baseline for harmonizing or validating higher resolution NICFI-Planet basemaps. The NASA Harmonized Landsat–Sentinel-2 dataset treats Sentinel-2 as the reference sensor for cross-calibration [29]. Similar approaches apply histogram- or regression-based adjustments to align PlanetScope or other sensor-derived reflectance with Sentinel-2 statistics [30,31]. Recent studies also compare and combine PlanetScope and Sentinel-2 reflectance for multi-sensor classification and validation tasks [32]. These works establish Sentinel-2 median mosaics as a reliable reference for cross-sensor harmonization.

In our present study, the Sentinel-2 median composite ($B_{i}^{S2,\mathrm{med}}$) serves as the reference against which high-resolution NICFI-Planet bands ($N_{i}$) are compared. We used only cloud-free scenes acquired within the same season as each NICFI-Planet acquisition to ensure comparable phenology and illumination. Note that here, band *i* is from the Sentinel-2 image, where i∈{1,2,3,4} which corresponds to bands B2 (Blue), B3 (Green), B4 (Red), and B8 (NIR).

First of all, we calculate per-band mean and standard deviation over all pixels in the median mosaic:

$$\mu_{i}\;=\;\frac{1}{\left|\Omega \right|}\sum_{(x,y)\in \Omega }B_{i}^{S2,\mathrm{med}}\left(x,y\right),\;\sigma_{i}\;=\;\sqrt{\frac{1}{\left|\Omega \right|}\sum_{(x,y)\in \Omega }{\left(B_{i}^{S2,\mathrm{med}}\left(x,y\right)-\mu_{i}\right)}^{2}}\;,$$

where Ω denotes the spatial domain of interest. $\mu_{i}$ is the mean of Sentinel-2 band *i* over the study domain and $\sigma_{i}$ is the standard deviation of Sentinel-2 band *i* over the same domain. In the above equations, (x,y) denotes the spatial coordinates of an individual image pixel within the study domain.

We then set each band’s threshold ($T_{i}$) as

$$T_{i}\;=\;\mu_{i}\;+\;\sigma \;\sigma_{i},$$

Here σ is a scalar multiplier controlling the sensitivity of the threshold with a default sensitivity scalar σ=1. This ensured that only anomalously bright (cloud-affected) pixels exceed this limit. For each NICFI-Planet pixel (x,y) and band *i*, a boolean mask $M_{i}\left(x,y\right)$ is generated:

$$M_{i}\left(x,y\right)\;=\;\left\{\begin{array}{cc} 1, & N_{i}\left(x,y\right)>T_{i},\\ 0, & \mathrm{otherwise}. \end{array}\right.$$

A pixel is retained if any band exceeds its threshold:

$$M_{\mathrm{combined}}\left(x,y\right)\;=\;\max_{i\in \{1,2,3,4\}}M_{i}\left(x,y\right)\;.$$

Here, $M_{\mathrm{combined}}\left(x,y\right)$ is the combined mask over all bands at pixel (x,y).

The final filtered NICFI-Planet bands are defined by

$$N_{i}^{\mathrm{filtered}}\left(x,y\right)=\left\{\begin{array}{cc} N_{i}\left(x,y\right), & M_{\mathrm{combined}}\left(x,y\right)=0,\\ \mathrm{masked}, & M_{\mathrm{combined}}\left(x,y\right)=1. \end{array}\right.$$

As mentioned above, algorithm B has a sigma value parameter (σ). We tried multiple σ values (0.5, 1, 2, and 3) to evaluate sensitivity of the output to this parameter. All computations, from mosaic creation through mask application, were executed directly in the GEE environment. Hence, we created a fully automated, reproducible workflow for cloud and anomaly masking of NICFI-Planet imagery using cloud-free Sentinel-2 statistics.

### 2.4. Supervised Classification for Comparative Analysis

We compared the outputs of the two approaches (Algorithm A and Algorithm B) against a Machine Learning (ML) algorithm. For this, We used a supervised Random Forest (RF) classifier, to assess whether simple, unsupervised methods could approximate supervised cloud detection without requiring labeled data. The RF was trained on the four NICFI-Planet bands using 500 decision trees. In addition to the NICFI-Planet bands, the RF received the corresponding Blue, Green, Red and NIR bands sampled from the Sentinel-2 median mosaics (as inputs) described in Section 3.1. Including Sentinel-2 mosaics provided a temporally matched, independently processed reference signal that has the potential to improve the classifier’s ability to discriminate cloud from dark surface features [33]. Although NICFI-Planet has finer native sampling than Sentinel-2, the Sentinel-2 mosaics furnish a seasonally matched, cloud-reduced baseline (via the median composites) and complementary sensor characteristics (different spectral response and atmospheric processing). Together, this multi-sensor approach increase robustness [33] to sensor-specific noise and acquisition artifacts and helped the RF generalize cloud signatures across sensors and seasons.

From a stratified random sample of 250 manually labeled pixels (125 cloudy or noisy, 125 clear), 70% were used for training and 30% for testing. Labeling was performed by a single annotator. RF hyperparameters were kept at default values.

## 3. Results and Discussion

### 3.1. Scene Selection

**Figure 2:**
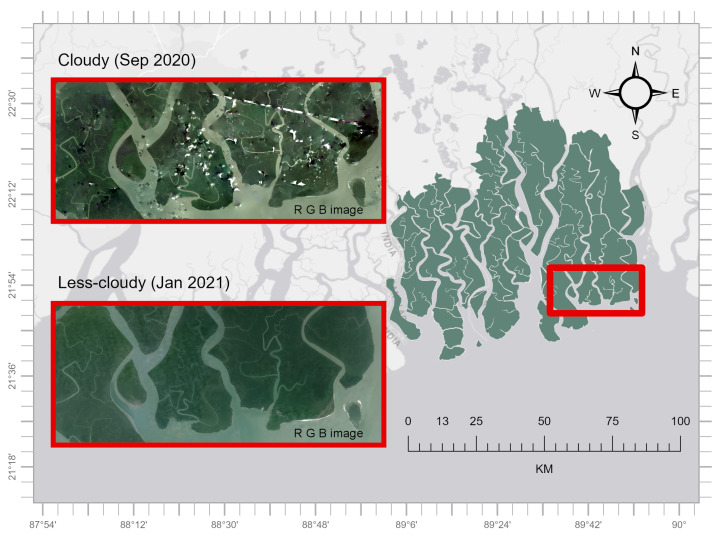
Two NICFI-Planet scenes from the study area used for testing the cloud-masking algorithms—one cloudier and one less cloudy. Polygons obtained from geoBoundaries [23] are used to delineate the forest boundary, which is displayed over ESRI Light Gray Canvas Map [34].

The study used two NICFI-Planet satellite images of the Sundarbans mangrove forest in Bangladesh for testing the proposed masking algorithms. One from September 2020 and another from January 2021. The image from September 2020 represents the late monsoon season and is characterized by substantial cloud cover common in tropical areas. The January 2021 image was chosen due to less cloud presence during the dry winter. This contrast helped to evaluate the performance of the proposed masking algorithm in conditions of both high and low cloud cover (Figure 2). We focused on an area (AOI) in Sundarbans mangrove forest where cloud cover and artifacts from the satellite data acquisition process were apparent.

For generating the Sentinel-2 reference mosaics, the selection of cloud-free scenes is automated based on the month and year of the target NICFI-Planet mosaic. The algorithm can be used to extract the month and year from the NICFI metadata and it then defines a compositing window around this period. The start date is set to the first day of the month preceding the target month, and the end date is set to the last day of the second month following the target month. The longer compositing window reflects seasonal constraints. The tropical study area with persistent cloud cover during the wet season necessitated aggregating several months of cloud-free pixels to obtain adequate spatial coverage across the AOI. Within this window, all available Sentinel-2 scenes are filtered for cloud-free pixels, and a 10 m median mosaic is generated in GEE. This automated selection ensures that the Sentinel-2 reference adapts dynamically to the temporal characteristics of each NICFI-Planet mosaic. It also avoids any need for manual scene selection while maintaining a basis for threshold computation.

For the monsoon (cloudy) scene the algorithm generated Sentinel-2 median mosaic from 18 cloud-free scenes acquired between 1 August and 30 November 2020. For the dry-season (less cloudy) scene, algorithm also selected 18 cloud-free scenes from the month of December 2020 to March 2021.

### 3.2. Cloud Detection with Algorithm A

**Figure 3:**
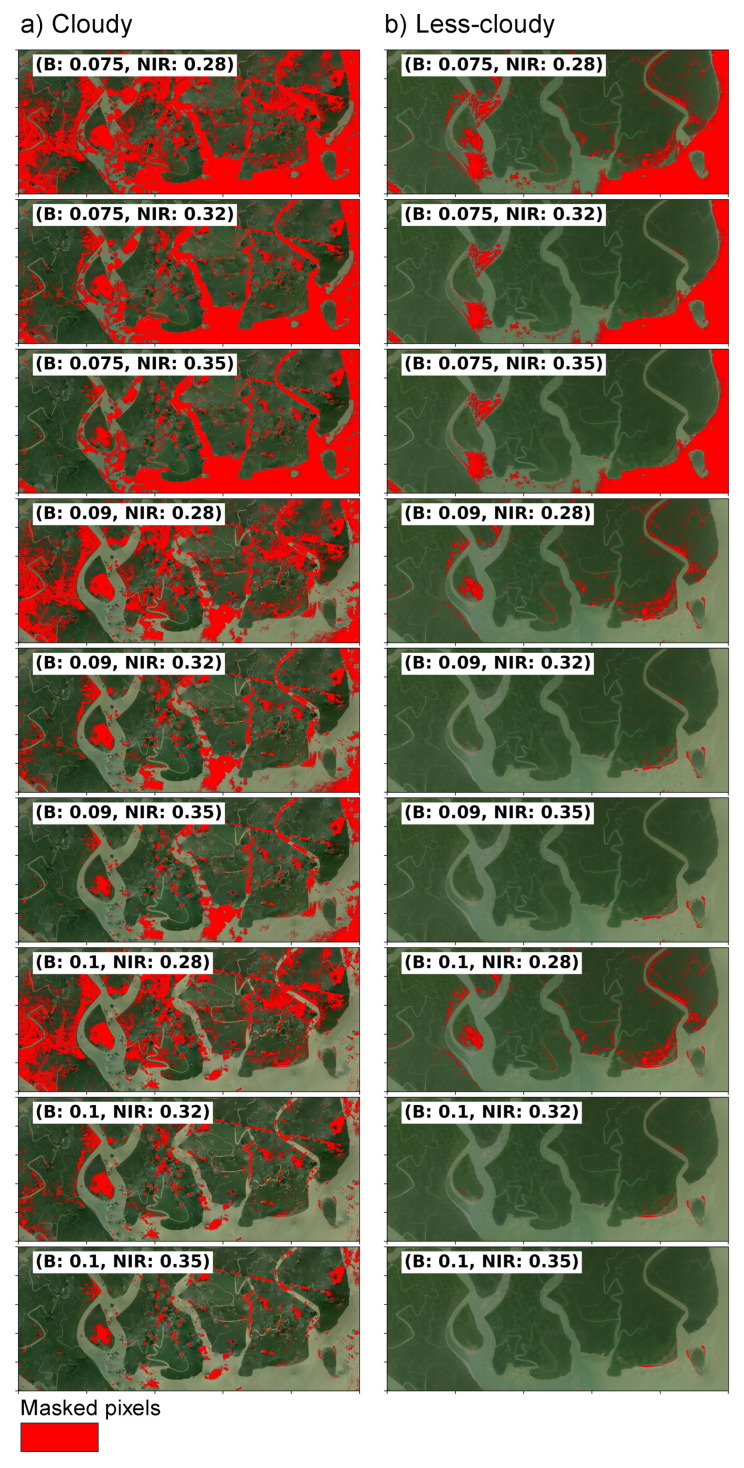
Results of experiments using algorithm A with multiple threshold values to mask clouds and other atmospheric interferences. Refer to Figure 2 for the original RGB images.

**Figure 4:**
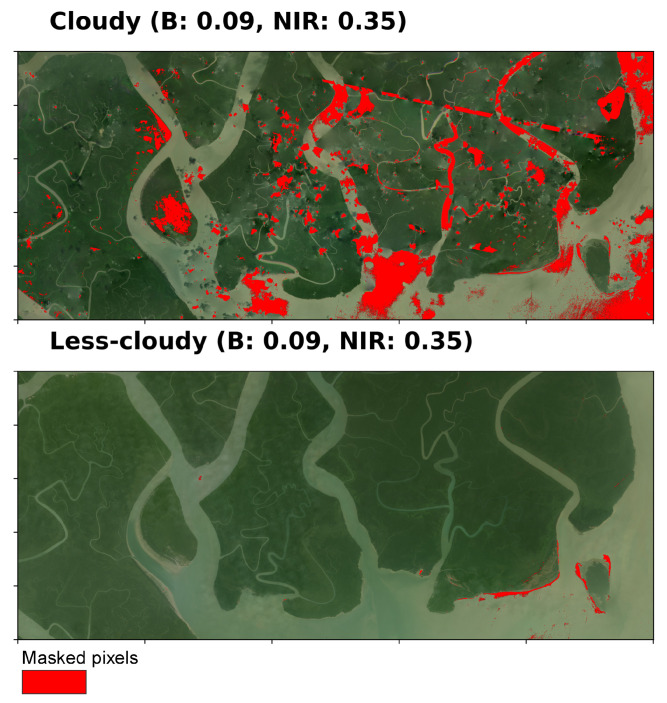
Result of flagging cloud and noise using algorithm A with thresholds of B: 0.09 and NIR: 0.35.

We present here the outcomes of applying Algorithm A for identifying cloud in NICFI-Planet scenes. The cloud detection algorithm underwent testing with nine distinct threshold sets, each aimed at identifying potential clouds and image artifacts (Figure 3). The threshold sets demonstrated varying intensities, from aggressive masking to moderate masking. The aim was to determine a good balance between cloud removal and the retention of usable pixels. Lower thresholds for the B and NIR bands (B: 0.075 and NIR: 0.28) resulted in an aggressive filter that effectively masked clouds while partially impacting non-cloudy pixels. Notably, the aggressive approach reduced haziness in the less cloudy image. As a byproduct, it also masked potential inland water pixels, which likely resulted from flooding or high tide events. Maintaining a constant Blue band threshold of 0.1 while incrementally raising the NIR threshold resulted in a moderated aggressiveness. This strategy demonstrated that an increased NIR threshold minimized masking but still reduced the presence of clouds, artifacts, and haze. Through this systematic observation and visual assessment, we identified a Blue band threshold of 0.09 and an NIR threshold of 0.35 as the most effective combination for this study area. We observe that in cloudy scene, Algorithm A (Figure 4) did not mask some small clouds and haze patches. However, the specified thresholds (B: 0.09; NIR: 0.35) appeared to reduce the influence of most clouds and artifacts, while also mitigating additional confounding variables such as haziness and water inundation. The latter holds particular significance in wetland regions, where atmospheric interference and forest flooding may generate misleading signals.

Note that the threshold values used for cloud and shadow detection can be modified according to the context of the image scene. Geographic region, seasonal variation, and atmospheric variables may influence the ideal values for these thresholds. Therefore, it is important to test different combinations of threshold values for a particular study area and come up with the ones that retain best pixels for further analysis.

### 3.3. Cloud Detection with Algorithm B

**Figure 5:**
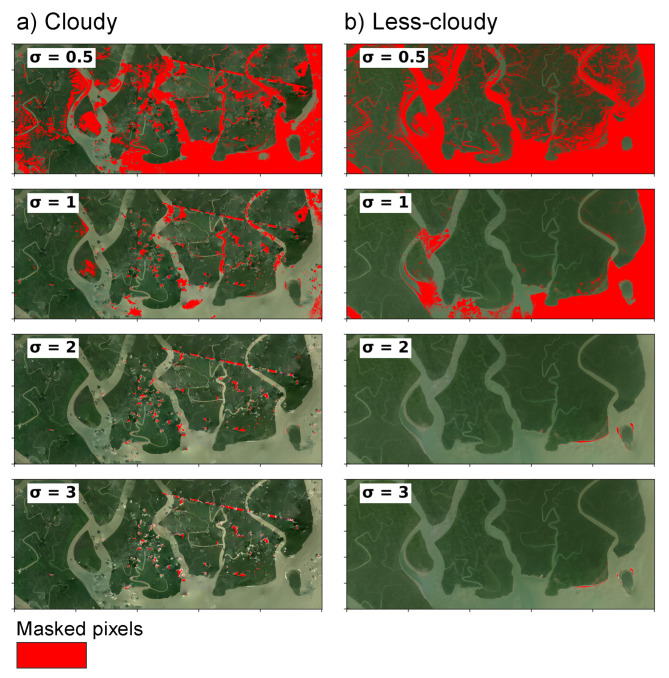
Results of experiments using algorithm B with multiple σ values to mask clouds and other atmospheric interferences. Refer to Figure 2 for the original RGB images.

The sensitivity of Algorithm B to the σ parameter was evaluated using a range of values (σ = 0.5, 1, 2, and 3). Across both heavily cloudy and relatively clear NICFI-Planet scenes, Algorithm B masked the major cloud and haze regions, as shown in Figure 5, where red pixels indicate the masked areas. At lower σ values, such as σ=0.5, the algorithm produces aggressive masking. It flags most potential cloud and haze but also over-mask usable pixels. At higher σ values, such as σ= 2 or 3, masking becomes more conservative, effectively covering the major cloud formations while keeping thin clouds unmasked. This reflects the the sensitivity of σ parameter. The statistical thresholding approach of Algorithm B leverages standard deviation cutoffs to avoid excessive masking of land and, therefore, is a critical consideration for ecological and land-cover monitoring.

**Figure 6:**
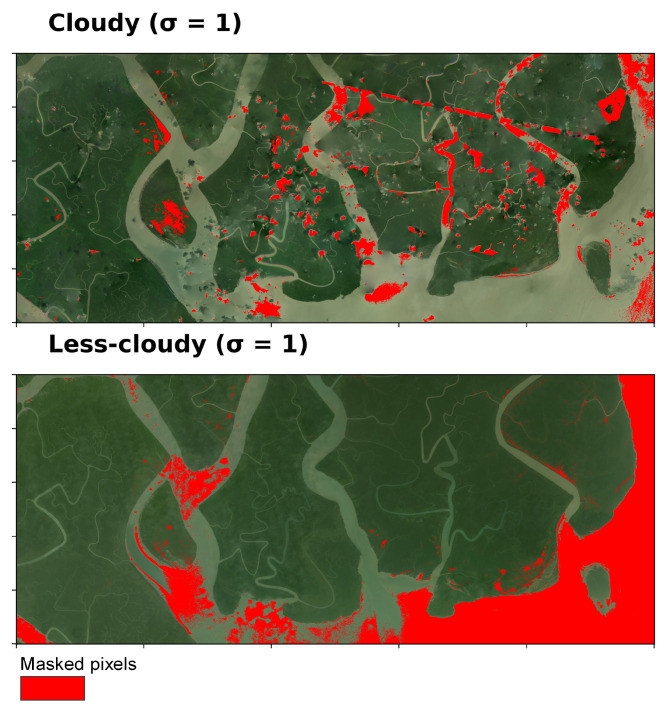
Result of flagging cloud and noise using algorithm B with σ=1.

We implemented Algorithm B with σ = 1 (Figure 6) to maximize retention of usable pixels in NICFI-Planet scenes while excluding clouds and haze. We observe that in cloudy scene, like Algorithm A, Algorithm B did not mask some small clouds and haze patches. Nevertheless, mangrove and non-mangrove classes remained distinguishable in these cases. We also note that the fixed thresholds provide simplicity and reproducibility; however, performance of Algorithm B may vary with atmospheric conditions, and an adaptive or multi-temporal approach could further reduce cloud omission in heavily overcast scenes.

### 3.4. Comparison of Algorithms with Supervised Machine Learning Based Classification

**Figure 7:**
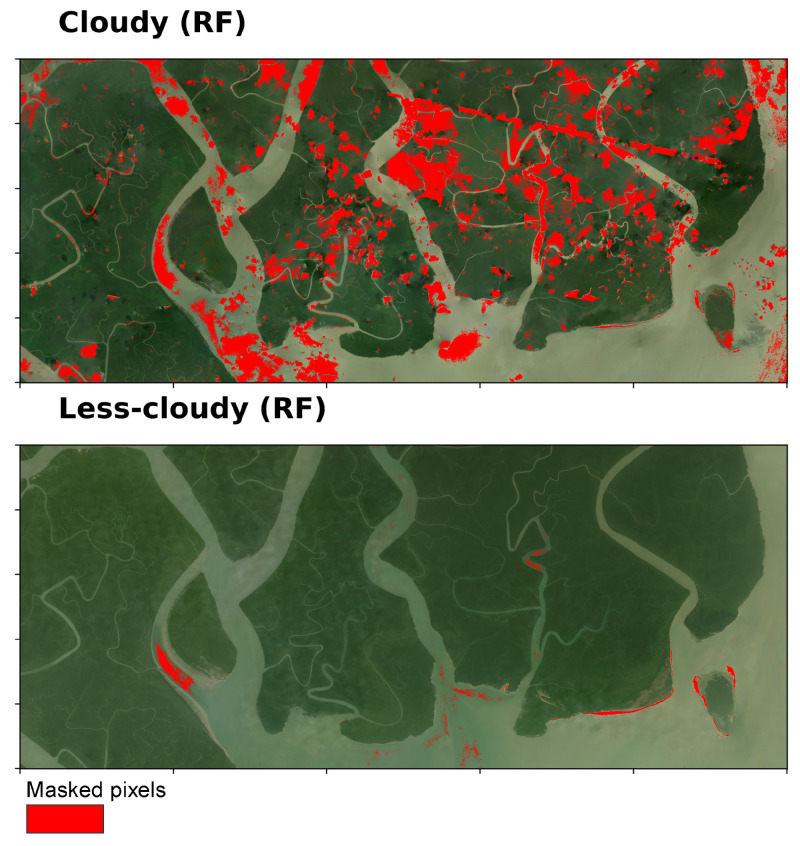
Cloud, noise, and haze masking using RF machine learning model.

We observed the results of the two methods (Algorithm A and Algorithm B) in comparison to RF (Figure 7). The RF model was trained primarily on cloudy scene and then was subsequently applied to less-cloudy scene. On the independent test partition, the classifier attained an overall accuracy of 78%.

**Figure 8:**
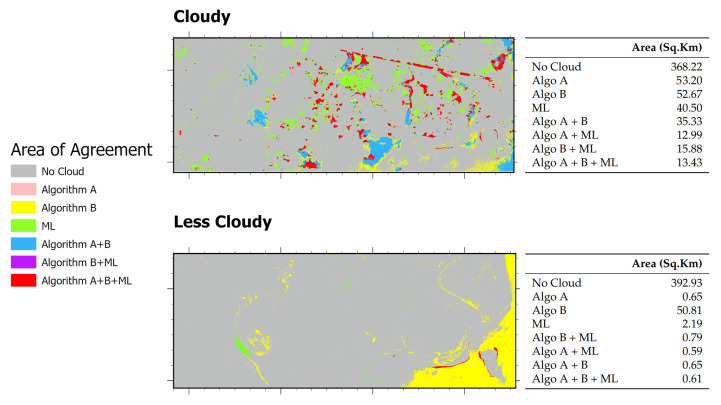
Area of Agreements in “cloudy” and “less-cloudy” scenes using Algorithm A, B, and ML. The maps show detection overlaps and individual contributions of each method.

A comparison of the spatial agreement among the three cloud-masking approaches (Algorithm A, Algorithm B, and the machine-learning (RF) method) across both cloudy and less-cloudy test scenes is presented in Figure 8. In the cloudy scene, Algorithm A detected the largest cloud-covered area (53.20 sq·km), closely followed by Algorithm B (52.67 sq·km) and the RF classifier (40.50 sq·km). The pairwise overlap between Algorithms A and B was 35.33 sq·km, the overlap between B and RF was 15.88 sq·km, and 13.43 sq·km were detected by all three methods. Visual inspection shows that A and B both capture extensive, continuous cloud regions and agree on a substantial core area, while RF identifies a somewhat smaller core but adds finer boundary delineation and small haze patches (Figure 7).

In the less cloudy scene (Figure 8), Algorithm B again returned the largest cloud area (50.81 sq·km; mostly flagging hazy water bodies as cloud/noise), whereas RF (2.19 sq·km) and Algorithm A (0.65 sq·km) detected very little. Nearly all pixels found by Algorithm A were also identified by Algorithm B and ML (A+B overlap ≈ 0.65 sq·km; three-way overlap ≈ 0.61 sq·km). This indicates that A contributes almost exclusively to the detection in the small core already captured by B. We observe that Algorithm B is highly sensitive—flagging light haze or bright water surfaces as cloud—while RF is comparatively conservative and Algorithm A offers only marginal additional detections in low-cloud conditions.

**Figure 9:**
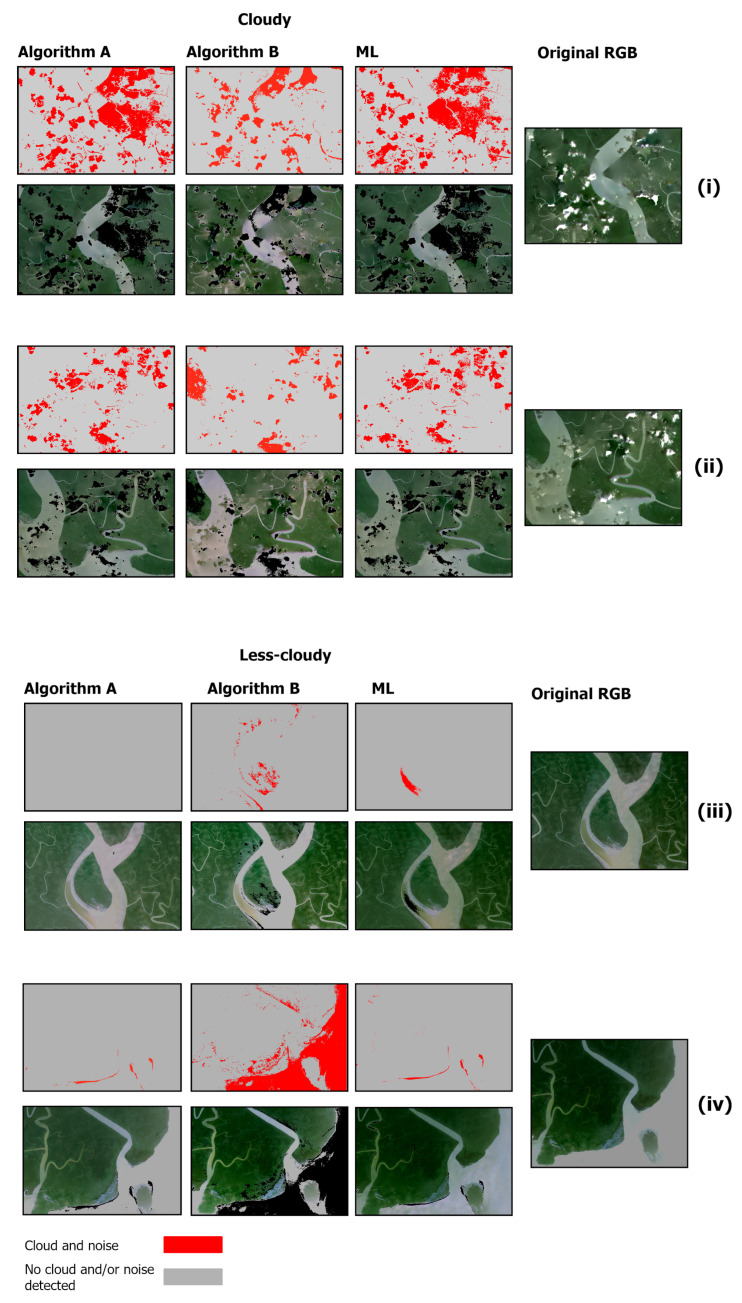
Zoomed examples of results comparing Algorithm A, Algorithm B, and the ML algorithm (RF classifier). Panels (**i**,**ii**) (cloudy scene) show low-contrast, hazy anomalies left unflagged by A and B but flagged by the RF. Panels (**iii**,**iv**) (less-cloudy scene) show RF and A agreeing with visual interpretation while Algorithm B is more aggressive.

The side-by-side comparisons of example cases also illustrate how Algorithm A, Algorithm B, and the ML algorithm respond to cloudy and less-cloudy conditions (Figure 9). In the cloudy scene, several patches (e.g., Figure 9i,ii) appear as low-contrast, hazy anomalies embedded within the dense inland mangrove canopy. Both Algorithm A and Algorithm B largely leave these patches unflagged (they remain classified as non-cloud), whereas the RF classifier flags many of these same pixels as cloud, shadow, or anomaly. This discrepancy was observed because the independent annotator labeled pixels from these regions as potential anomalies (cloud/shadow) and the RF therefore learned to treat that spectral signature as cloud/shadow suspect. We cannot, however, assert with confidence that the RF labels are true clouds or shadows. Based on spectral appearance and local context, three plausible explanations exist. (1) Transient flood inundation (these tidal/monsoon zones are frequently inundated and waterlogged canopy or standing water can alter reflectance); (2) image-acquisition artifacts (sensor noise, sun-glint, or local processing artifacts); or (3) true cloud/noise/shadow. At present the data do not uniquely discriminate among these causes, so it is recommended to treat the RF detections in these patches as plausible but unresolved anomalies rather than confirmed cloud.

In the less-cloudy scene, the algorithms perform more consistently (e.g., Figure 9iii,iv). The results from RF and Algorithm A align with our visual interpretation, while Algorithm B is noticeably more aggressive and flags additional hazy/bright water pixels as cloud. We hypothesize that these extra detections by Algorithm B are consistent with commission errors caused by low-optical-depth haze or bright water/sun-glint being mistaken for cloud.

Considering both scenes together, Algorithm B is the most aggressive detector overall for less cloudy scenes, RF is moderate and adaptive, and Algorithm A is generally conservative. Thus, in our study area context, Algorithm B is preferable when maximizing cloud removal is the priority (particularly for sparse or subtle clouds), RF is advantageous when labeled training data are available and a balance between omission and commission is required, and Algorithm A is appropriate where minimizing loss of surface pixels is critical. For large-scale automated processing without labeled data, Algorithms A and B remain attractive for their simplicity and scalability, with the caveat that Algorithm B may overestimate clouds in less-cloudy imagery.

### 3.5. Spectral Ambiguity Between Cloud Shadows and Forest Canopy

Spectral ambiguity between cloud shadows and submerged forest canopy (in a coastal wetland) creates a substantial challenge for the human interpreter of optical remote-sensing pixels. Both cloud shadows and dense canopies adjacent to river, creeks and water bodies in our cloudy scene appear darker in visible and NIR owing to similarly low reflectance. We hypothesize this is partly caused by mangroves being routinely inundated during monsoon high tides, which reduces canopy reflectance and increases the likelihood of confusing submerged canopy with cloud shadow, contributing to misclassification in automated land-cover mapping and change detection [35]. The problem is compounded in areas with complex landscapes or heterogeneous vegetation cover, as variation in solar illumination, canopy structure, and topography also affect the spectral response and further obscure the boundaries between these features. To address these challenges, several alternative methods can be adopted, such as geometry-based cloud shadow detection (considering sun-cloud-sensor geometry), the use of multi-temporal imagery to distinguish persistent features from transient shadows, and the application of deep learning models trained to identify and correct for uneven illumination [35,36].

Hyperspectral data could also offer a promising solution in this context. Because hyperspectral sensors collect reflectance information across many narrow spectral bands, they can more precisely characterize subtle spectral differences between shadowed canopy and true cloud shadows [37,38,39]. Previous studies using hyperspectral imagery have detected shadows by exploiting spectral signatures around specific wavelengths that show greater contrast between sunlit and shaded vegetation [37]. While such data are not used in our current study, we acknowledge that future work leveraging hyperspectral acquisition could significantly improve separation of cloud shadows from forest canopy.

### 3.6. Applicability Across Two Other Deltaic Regions

**Figure 10:**
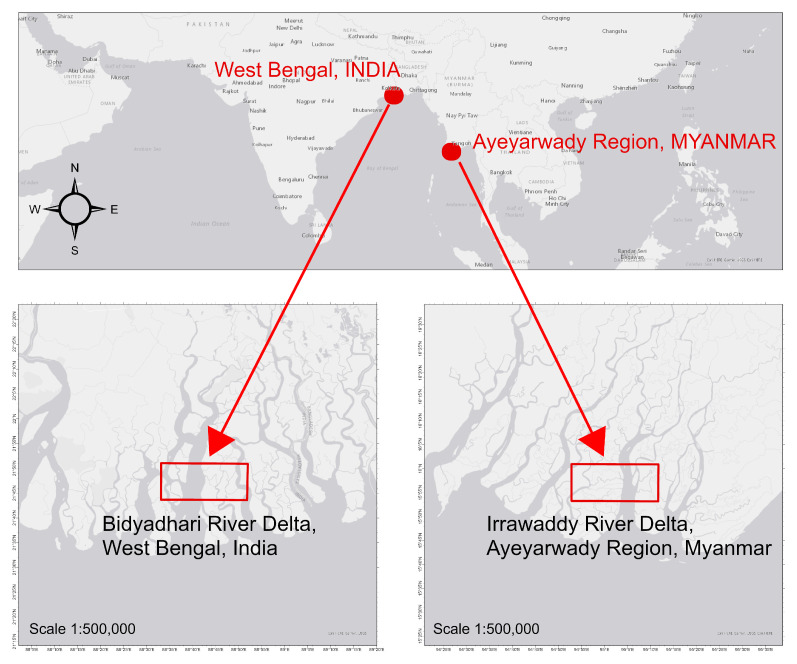
Locations of the two study sites: the Bidyadhari River Delta, West Bengal, India, and the Irrawaddy River Delta, Ayeyarwady Region, Myanmar, displayed over ESRI Light Gray Canvas Map [34].

We expanded our study to include two ecologically and hydrologically distinct mangrove deltaic systems: (i) the Bidyadhari River Delta, West Bengal, India, and (ii) the Irrawaddy River Delta, Ayeyarwady Region, Myanmar (Figure 10). To ensure that our results generalize beyond a single local context, these locations were selected to test our algorithms. Both sites have similar regional features as our original study site, including a tropical monsoon climate, strong tidal influence, and deltaic plain [40,41,42,43]. The Bidyadhari delta is a freshwater-starved, salinity-stressed fringe of the Indian Sundarbans, shaped by upstream flow regulation and chronic dry-season hypersalinity [41]. In contrast, the Irrawaddy Delta historically receives one of the largest freshwater and sediment loads in the region [42,43].

**Figure 11:**
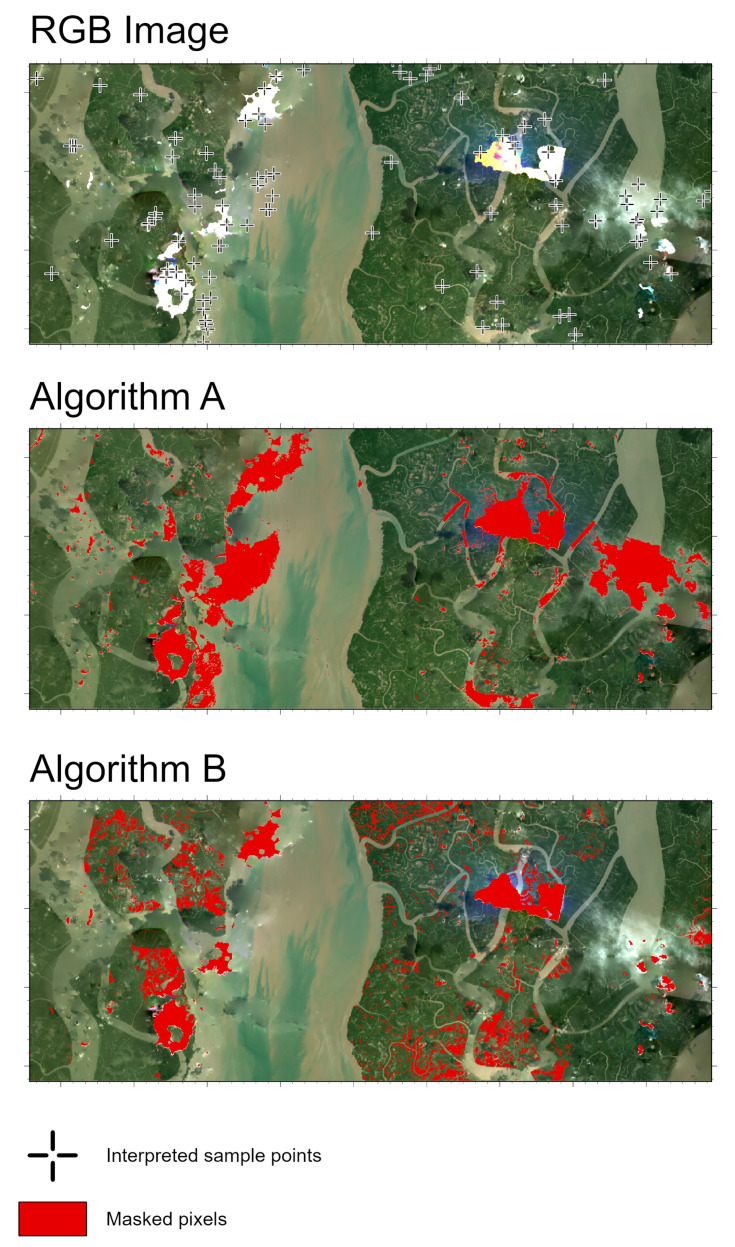
RGB NICFI-Planet image and corresponding results from Algorithm A and Algorithm B for Bidyadhari River Delta, West Bengal, India. Red pixels indicate masked cloud, noise or haze regions, and crosses on RGB image denote visually interpreted reference points.

**Figure 12:**
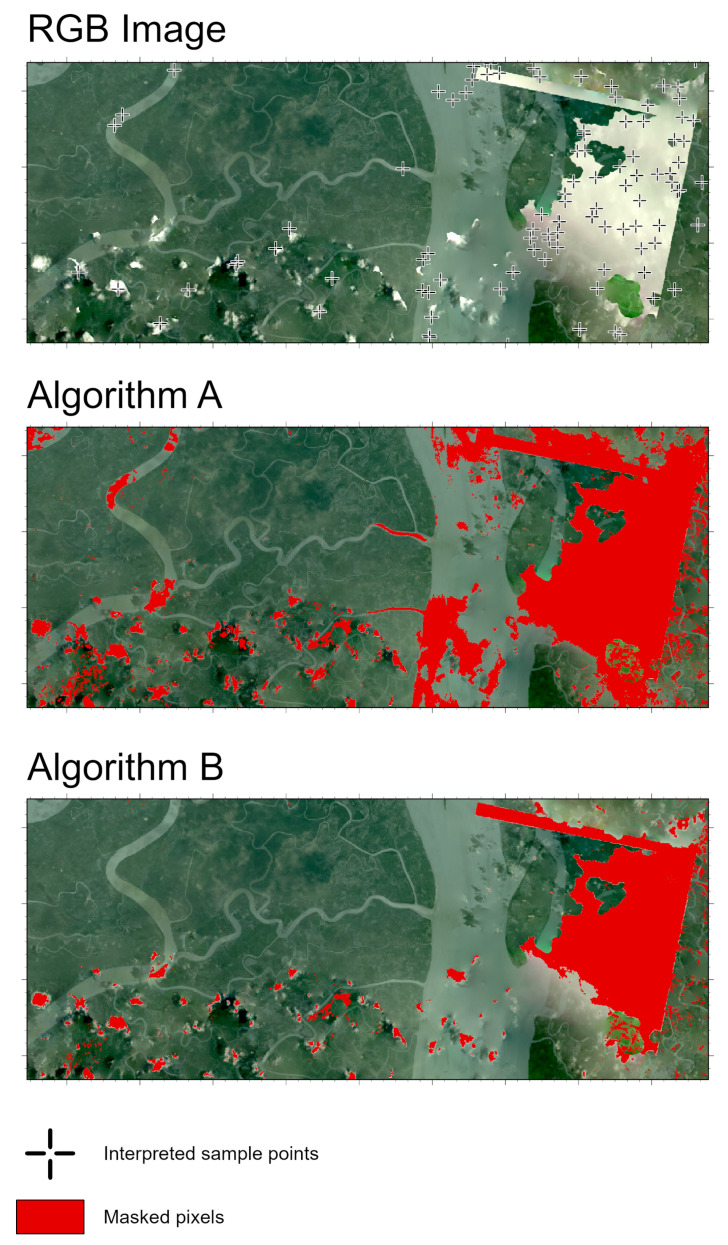
RGB NICFI-Planet image and corresponding results from Algorithm A and Algorithm B for Irrawaddy River Delta, Ayeyarwady Region, Myanmar. Red pixels indicate masked cloud, noise or haze regions, and crosses on RGB image denote visually interpreted reference points.

Across both sites, Algorithm A shows a more aggressive masking behavior and was able to flag extensive regions affected by haze, low-contrast cloud streaks, and even turbid water bodies (Figure 11 and Figure 12). This is visually evident in the dense and spatially widespread masked outputs. The aggressiveness of Algorithm A suggests a higher sensitivity to brightness anomalies which essentially is beneficial for capturing subtle haze but accompanied by a higher rate of false positives. In contrast, Algorithm B systematically produces more conservative masks. It flags only the higher-intensity cloud features while leaving large areas of low-reflectance haze and near-shadow conditions unmasked. This results in spatially sparse patches, with significant portions of visually apparent thin cloud remaining unflagged.

To assess performance on these two new sites, we first merged all pixels flagged by Algorithm A and Algorithm B to form a unified mask of potentially anomalous areas. Within this masked region, we then randomly generated 100 points per site (Figure 11 and Figure 12). An independent interpreter, who was blinded to whether a pixel had been flagged by either algorithm, evaluated each point to determine whether it represented a true anomaly. This approach provided an unbiased estimate of algorithmic accuracy while ensuring that the assessment focused specifically on areas deemed high-likelihood by at least one method.

The analysis of cloud-masking algorithms A and B, based on 100 interpreted pixels over the Bidyadhari River Delta, West Bengal, India, shows some performance disparities (Table 1). In terms of accurately identifying actual cloud cover, referred to as the True Positive (Recall) rate, Algorithm A correctly classified 50 of the 55 actual cloud pixels, achieving a high recall of 90.91%.Conversely, Algorithm B demonstrated poor sensitivity, only identifying 23 cloud pixels, resulting in a significantly lower recall of 41.82%. Algorithm A was far more effective at minimizing the false positive rate (incorrectly flagging a clear area as cloudy). Algorithm A recorded 10 false positives, which is 30.30% of the 33 actual clear-sky samples. Algorithm B showed an inflated false positive rate, with 24 false positives, or 72.73%.

The analysis of the cloud-masking algorithms over the Irrawaddy River Delta, Ayeyarwady Region, Myanmar reveals similar performance (Table 2). Again, algorithm A shows an aggressive detection pattern, correctly identifying all 78 actual cloud pixels for a perfect True Positive (Recall) rate of 100.00%, but simultaneously classifying all 14 clear-sky pixels as cloudy. Conversely, Algorithm B displays highly conservative behavior and avoided all false positive with a 0.00% rate, but misses a number of actual clouds, achieving a lower Recall of 64.10% (50 out of 78).

In short, we observe that the aggressiveness of the algorithms are region-dependent. It appears more conservative in the Sundarbans but noticeably more over-inclusive in the India and Myanmar scenes. Algorithm A is prone to over-masking but reliably captures subtle noise/haze/cloud, while Algorithm B avoids over-masking but misses thin clouds and diffuse haze. It is important to note that Algorithms A and B were not specifically tuned for the Bidyadhari or Irrawaddy Delta sites. Despite this, both algorithms were still able to flag potential clouds and haze effectively across ecologically distinct mangrove systems. We hypothesize that optimizing the threshold values for these sites could further improve detection performance. Once appropriate thresholds are established for a given location, the algorithms can be applied consistently over temporal series of NICFI-Planet mosaics for any years and months, simply by mapping the algorithm across the image collection in GEE, without additional manual intervention or training samples for each scene.

### 3.7. Limitation

Our results are promising, however, the present work has several limitation. First, for RF, the training samples were collected from cloudy scene, and model behavior when applied to less-cloudy imagery may, therefore, differ from its behavior on the training distribution. Second, interpreter’s ambiguity was non-trivial. The independent image interpreter encountered pixels with diffuse haze that could plausibly be explained by cloud, by image-processing artifacts, or by land being temporarily submerged (tide), and we do not have independent ground truth to disambiguate these cases. Consequently, the reported overall accuracy should be regarded as indicative of performance on the labeled dataset rather than a definitive estimate of absolute accuracy under broader field conditions. The small reference dataset (250 pixels) and single-annotator labeling limit the statistical strength of the evaluation. Future work should expand the labeled dataset, incorporate multi-temporal filtering, explore adaptive thresholds, and evaluate the use of thermal bands, QA layers, or deep-learning-based cloud detection. Future work should also quantify inter-annotator agreement, incorporate ancillary data (e.g., tidal records, higher-resolution imagery), and consider uncertainty-aware or consensus labeling to reduce ambiguity.

## 4. Conclusions

This study evaluated two threshold-based cloud-masking algorithms, Algorithm A and Algorithm B, on NICFI-Planet basemaps of the Sundarbans mangrove forest. Performance was first assessed on two representative scenes with contrasting atmospheric conditions: one highly cloudy and one relatively clear. Algorithm B exhibited a more aggressive masking behavior, effectively capturing subtle haze, low-contrast cloud streaks, and turbid water, though at the cost of occasional over-masking. Algorithm A, in contrast, was more conservative, detecting only the higher-intensity cloud features while leaving low-reflectance haze and near-shadow areas unflagged. These complementary behaviors highlight the trade-off between sensitivity and specificity, allowing users to select the algorithm that best suits their processing needs.

To assess applicability beyond the original site, both algorithms were tested in two additional deltaic regions: the Bidyadhari River Delta (India) and the Irrawaddy River Delta (Myanmar). Analysis of random reference points in these sites showed that Algorithm A consistently achieved high recall, capturing subtle cloud and haze features, while Algorithm B avoided over-masking but missed thinner clouds. These results demonstrate that the algorithms maintain consistent performance across ecologically and hydrologically distinct mangrove deltaic systems. However, the aggressiveness of the algorithms varies by region. For example, algorithm A is comparatively conservative in the Sundarbans yet substantially more over-inclusive in the India and Myanmar scenes. It is worth noting that Algorithms A and B were not specifically tuned for these two locations; nevertheless, they were able to flag potential cloud areas. Optimizing the threshold values for each site could further improve performance, and once tuned, the algorithms can be applied consistently across temporal series simply by mapping over the image collection.

These two algorithms offer practical advantages: they are easy to implement in GEE, fully automatic, and do not require any manual labeling or training data. All source codes have been made openly available (see Supplementary Materials) to ensure transparency and reproducibility. This combination of effectiveness, simplicity, and reproducibility demonstrates their utility compared to existing approaches, particularly for large-scale, low-overhead processing of NICFI-Planet collections. While a supervised Random Forest classifier was included as a reference, its role was secondary. ML models require labeled training data and extensive tuning, whereas Algorithms A and B provide transparent, scalable, and operationally practical solutions. Overall, both Algorithm A and B provide a first-pass masking solution in cloudy and hazy conditions.

## Figures and Tables

**Table 1:** Comparison of cloud flagging of algorithm A and B vs. interpreter’s labeling on 100 Sample Points in India.

Interpreter’s Label	Algorithm A		Algorithm B	
	Cloud	No Cloud	Cloud	No Cloud
Cloud	50	5	23	32
Unsure	8	4	4	8
No Cloud	10	23	24	9

**Table 2:** Comparison of cloud flagging of algorithm A and B vs. interpreter’s labeling on 100 Sample Points in Myanmar.

Interpreter’s Label	Algorithm A		Algorithm B	
	Cloud	No Cloud	Cloud	No Cloud
Cloud	78	0	50	28
Unsure	8	0	2	6
No Cloud	14	0	0	14

## Data Availability

All Google Earth Engine codebooks are available here: https://github.com/kmashraful/nicfi_planet_cloud_filtering_repo (accessed on 2 October 2025).

## Supplementary Materials

The following supporting information can be downloaded at: https://www.mdpi.com/article/10.3390/s25247559/s1, Supplementary Materials: All GEE codes.
