# Supplementary material for: Two Novel Cloud-Masking Algorithms Tested in a Tropical Forest Setting Using High-Resolution NICFI-Planet Basemaps
Source: Sensors (Basel). 2025 Dec 12;25(24):7559. doi: 10.3390/s25247559 (PMC12737095; doi:10.3390/s25247559)
Supplement: Supplementary file 1 [file sensors-25-07559-s001.zip › sensors-3938069-supplementary.pdf]

Algorithm A: Threshold based cloud filtering raw GEE codebook:
https://code.earthengine.google.com/a2e63af30378ed620f4e54f22e616f56

Algorithm B: Stat-thresholding based cloud filtering raw GEE codebook:

https://code.earthengine.google.com/02e71ce9205920d9936b662bea784219

ML Model Development Raw:

https://code.earthengine.google.com/1f0884f760b076e30a1e70a8b6a701e7

GEE ASSET IDs:

Less cloudy NICFI-Planet Scene:

projects/ee-islamkm/assets/noncloudy_nicfi_unfiltered

Cloudy NICFI-Planet Scene:

projects/ee-islamkm/assets/cloudy_nicfi_unfiltered

Github repo: https://github.com/kmashraful/nicfi_planet_cloud_filtering_repo
